# Changes in educational inequalities in knee and hip osteoarthritis surgery and non-surgery specialist care visits over time in Sweden

**DOI:** 10.1016/j.ocarto.2024.100470

**Published:** 2024-04-11

**Authors:** Maria Lindéus, George Peat, Martin Englund, Ali Kiadaliri

**Affiliations:** a Lund University, Faculty of Medicine, Department of Clinical Sciences Lund, Orthopaedics, Clinical Epidemiology Unit, Lund, Sweden; b Lund University, Centre for Economic Demography, Lund, Sweden; c Centre for Applied Health & Social Care Research (CARe), Sheffield Hallam University, Sheffield, South Yorkshire, UK; d Primary Care Centre Versus Arthritis, School of Medicine, Keele University, Keele, Staffordshire, UK

**Keywords:** Osteoarthritis, Education, Inequality, Concentration index, Blinder–oaxaca decomposition

## Abstract

**Objective:**

To examine changes in prevalence and socioeconomic inequalities in knee and hip OA outcomes, in more specific surgery and non-surgery specialist care visits, from 2001 to 2011 in Sweden and to what extent sociodemographic factors can explain the changes.

**Design:**

We included all individuals aged ≥35 years resident in Sweden from 2001 to 2011. Individual-level data was retrieved from the Swedish Interdisciplinary Panel. Highest educational attainment was used as socioeconomic measure and the concentration index was used to assess relative and absolute educational inequalities. We used decomposition method to examine changes in prevalence and relative educational inequalities.

**Results:**

A total of 4,794,693 and 5,359,186 people were included for the years 2001 and 2011, respectively. The crude prevalence of surgery and specialist visits for knee and hip OA was 36–83% higher in 2011 than in 2001. The increase in hip OA outcomes was largely explained by changes in the sociodemographic composition of the population, whereas for knee OA outcomes, changes in the strength of the associations with sociodemographic factors appeared more important. All outcomes were concentrated among people with lower education in all study years. The relative inequalities declined over the study period, while the absolute inequalities increased for knee OA outcomes and remained stable for hip OA.

**Conclusion:**

Our findings show an increasing burden of all studied OA outcomes. Moreover, our findings suggest persistent educational inequalities with more surgeries and specialist visits among lower-educated individuals. Future research should incorporate additional variables to better understand and address these inequalities.

## 1 Introduction

Osteoarthritis (OA) is the most common joint disorder [1] and is a major cause of disability [2,3]. According to the Swedish National Board of Health and Welfare's national guidelines, the basic treatment of OA should consist of patient education, exercise, and weight control [4]. Surgical intervention is an option if basic and adjunctive treatments (e.g. pharmacological treatment of pain) have not produced sufficient effect [4]. About 30,000 knee and hip surgeries were performed due to OA in Sweden in 2019 [5,6].

Numerous studies have examined the association between sociodemographic factors and OA-related outcomes [[7], [8], [9], [10], [11], [12], [13], [14], [15], [16], [17], [18], [19]]. Previous research has reported an inverse association between various measures of socioeconomic status and risk of OA [9,11,12,14,15], severity/impact of OA [10,11,13,16], and rate of knee arthroplasty [17]. Although research has shown socioeconomic inequalities in OA, little is known about factors contributing to these inequalities and their changes over time in Sweden.

We aimed to examine changes in prevalence and socioeconomic inequalities, measured by the concentration index, in knee and hip OA surgery and non-surgery specialist care visits from 2001 to 2011 in Sweden using national register data and decomposition analysis. Decomposition of socioeconomic inequality has never been explored in the OA context and by using decomposition analysis it is possible to assess different factors contribution to potential changes in inequality between two time points. We aimed to answer the following research questions: 1) How has the prevalence of knee and hip OA surgery and non-surgery specialist care visits changed and to what extent can sociodemographic factors explain the changes? 2) How have educational inequalities in knee and hip OA surgery and non-surgery specialist care visits developed and what factors contributed to this pattern?

## 2 Method

We included all individuals aged ≥35 years resident in Sweden during years 2001–2011, treating each year as a cross-section. However, when decomposing the changes of prevalence and educational inequalities of the outcomes, only the year of 2001 and 2011 were compared with each other.

### 2.1 Data sources

The data used in this paper comes from the Swedish Interdisciplinary Panel (SIP) approved by the Regional Ethics Committee in Lund (2012/03). The SIP contains information from several national registers and the registers are linked with each other by the personal identification number assigned to all residents in Sweden. We collected individual-level data from the following registers in SIP: The Total Population Register (year of birth, sex, civil status, country of birth), the Longitudinal integration database for health insurance and labour market studies (highest educational attainment, disposable income, employment), The Swedish National Patient Register (information on outpatient specialist care OA diagnosis and OA surgery). The two former registers are maintained by the Statistics Sweden while the latter is maintained by the Swedish National Board of Health and Welfare. The Swedish National Patient Register covers outpatient specialist care visits including day surgery from both public and private caregivers and the data is available from 2001. The register does not include data concerning primary care visits (https://www.socialstyrelsen.se/en). The year 2011 was selected for comparison with 2001 because certain variables in the SIP only had data up to and including that year.

### 2.2 Sociodemographic variables

We used the individual's highest educational attainment as our socioeconomic measure. This was transformed into years of education with a range from 7 to 20 years, where 7 years represented the most basic education and 20 years represented PhD education (supplementary material, Table A1). The decision to include people of 35 years and older was based on the assumption that the majority of people at this age have attained their highest educational level. We included the following sociodemographic variables in our analyses: sex (female ​= ​0, male ​= ​1), age (as a continuous variable), country of birth (Sweden-born ​= ​0, foreign-born ​= ​1), civil status (never married ​= ​0, married/registered partnership ​= ​1, previously married/in a partnership ​= ​2), employment (unemployed or not in the labour force ​= ​0, employed ​= ​1), disposable income (five groups based on quintiles), highest educational attainment of mother and father, respectively (≥10 years of education ​= ​0, <10 years of education ​= ​1, missing education ​= ​2), country of birth of parents (both parents born in Sweden ​= ​0, at least one foreign-born parent ​= ​1, missing parents' country of birth ​= ​2). We only had missing data on income (n ​= ​45 in 2001 and n ​= ​1 in 2011), educational attainment of mother (51.7% in 2001 and 39.1% in 2011), educational attainment of father (64.3% in 2001 and 50.7% in 2011), and parents' country of birth (34.1% in 2001 and 25.0% in 2011). For parents' education and country of birth the missing data was included as a sub-category, since there was not enough information to use imputation. We excluded those with missing income since the sample size was small.

### 2.3 OA surgery and non-surgery specialist care visits

Knee and hip OA surgery were defined as knee (ICD-10 codes: M17) or hip OA (ICD-10 codes: M16) registered as the main diagnosis at the same time as a knee or hip OA-related surgical procedure (arthroplasty or osteotomy) was registered. For knee OA surgery we used the following surgery codes: NGB49, NGB19, NGK59 NGB29, NGB59, NGB53, NGB09, and NGB39. Following surgery codes were used for hip OA surgery: NFB49, NFB29, NFB39, NFB99, NFK59, and NFB62. The surgery codes are based on the National Board of Health and Welfare's classification of surgical procedures [20] (see supplementary material, Table A2). Knee and hip OA non-surgery specialist care visits were defined as knee (ICD-10 codes: M17) or hip OA (ICD-10 codes: M16) registered as the main diagnosis without OA-related surgical procedure registered at the same time. A person with surgery wasn't counted as a non-surgery specialist care visit. If an individual had a surgery as well as a non-surgery specialist care visit the same year, that person was counted as both surgery and non-surgery specialist care visit.

People with more than one OA surgery and/or non-surgery specialist care visit in a year were only counted once in that year.

### 2.4 Statistics

We computed the concentration index to quantify the magnitude of educational inequality in knee and hip OA surgery and non-surgery specialist care visits. The concentration index is derived from the concentration curve, which in turn is generated by plotting the cumulative proportion of the population (X-axis) ranked by socioeconomic measure against the cumulative proportion of the desired outcome variable (y-axis) [21,22]. If there is no educational inequality in outcome, the concentration curve will be a 45-degree line, running from the bottom left-hand corner to the top right-hand corner, called the line of equality. The concentration index is defined as twice the area between the concentration curve and the line of equality. The index has an interval from −1 to 1, where a negative (positive) value indicates that the outcome is disproportionately concentrated among people with low (high) educational attainment. When the y-axis represents the share of the outcome variable the results represent the Relative Concentration Index. Absolute Concentration Index is defined as follows: ACI ​= ​MEAN∗RCI [21,22]. To provide a more comprehensive picture of socioeconomic inequalities [23], we quantified both relative and absolute inequalities which may show different direction of change and magnitude and therefore lead to different conclusions. Relative measurements of inequality state that inequality would not change if every member of a population experienced a change in health that was the same relative amount. On the other hand, absolute measurements of inequality state that inequality would remain constant if every member of a population experienced a change in health of the same absolute magnitude. In order to take the bounded and binary nature of the outcome variables into account, we employed the Wagstaff Index (WI) [24] to estimate the Relative Concentration Index. To estimate the WI and the ACI, the *conindex* function in Stata was used [25].

We used the Blinder–Oaxaca decomposition method [26,27] to examine changes in both prevalence and relative educational inequalities of the four outcomes (knee and hip OA surgery and knee and hip OA non-surgery specialist care visits) between the year of 2001 and 2011. The method enabled us to assess to what extent the changes between the two years can be explained by changes in the selected sociodemographic characteristics of the populations, i.e. the *explained* part of the decomposition, versus differences in the coefficients of these sociodemographic characteristics, i.e. the *unexplained* part of the decomposition [26,27]. The unexplained part of the decomposition also contains unmeasured factors which are described as a constant. Regarding the examination of prevalence, the *oaxaca* function in Stata was used and given the binary outcome we used logit model [28]. To examine changes in educational inequality, we used Recentered influence function (RIF) decomposition [29]. RIF is a statistical tool used to calculate the extent each individual influences the statistics of interest [29]. The RIF decomposition method decomposes the concentration index, in more specific the WI in our study, by using RIF regression. For these analyses we used the *oaxaca_rif* function in Stata [29]. We computed the decomposition from the perspective of the population of 2011 with the population of 2001 as reference. Thus, we examined what the prevalence and inequalities would have been if the population of 2011 would have had the same characteristics and coefficients of the selected sociodemographic variables as the population of 2001. Stata version 17.0 was used for the analyses.

## 3 Results

A total of 4,794,693 (48.6% females) and 5,359,186 (48.9% females) people were included for the years 2001 and 2011, respectively. The mean (SD) age was 55.8 (13.9) and 57.3 (14.4) years in 2001 and 2011, respectively. The proportion of the study sample with >12 years of education was 25.6% and 32.2% in 2001 and 2011, respectively. In 2001, there were 5628 knee OA and 7895 hip OA surgeries and the corresponding numbers for 2011 were 11,477 and 12,442, respectively. For non-surgery specialist care visits, there were 17,360 visits with knee OA as the main diagnosis in 2001 and 8988 for hip OA at the same year. Corresponding numbers were 33,184 and 13,688, respectively, in 2011 (Table 1). Characteristics of the study populations of 2001 and 2011 are presented in Table 1. Descriptive statistics for the study populations for all years between 2001 and 2011 are presented in table A3 in the supplementary material.

**Table 1:** Descriptive statistics of the study populations of the years 2001 and 2011.

	2001	2011
Number of people (n)	4,794,693	5,359,186
Number of knee OA surgeries (n)	5628	11,477
Prevalence per 100,000 people	117	214
Number of hip OA surgeries (n)	7895	12,442
Prevalence per 100,000 people	165	232
Number of non-surgery visits in specialist care with knee OA as main diagnosis (n)	17,360	33,184
Prevalence per 100,000 people	362	619
Number of non-surgery visits in specialist care with hip OA as main diagnosis (n)	8988	13,688
Prevalence per 100,000 people	187	255
Mean age ​± ​SD	55.8 ​± ​13.9	57.3 ​± ​14.4
Females (%)	48.6	48.9
Foreign-born (%)	12.8	15.6
Missing foreign-born (%)	0	0
Sweden-born parents (%)	57.0	64.4
Missing (%)	34.1	25.0
Educational attainment		
> ​12 years of education (%)	25.6	32.2
Missing (%)	0	0
Educational attainment of mother		
<10 years of education (%)	30.6	31.9
Missing (%)	51.7	39.1
Educational attainment of father		
<10 years of education (%)	20.2	24.2
Missing (%)	64.3	50.7
Income		
Mean disposable income (SEK)	163,276	244,115
Missing (n)	45	1
Civil status		
Married (%)	56.7	52.9
Previously married (%)	24.6	24.1
Never married (%)	18.8	23.1
Missing (%)	0	0
Employment		
Employed (%)	58.0	58.0
Missing (%)	0	0

### 3.1 Secular trends in prevalence of surgeries and specialist care visits

**Fig. 1:**
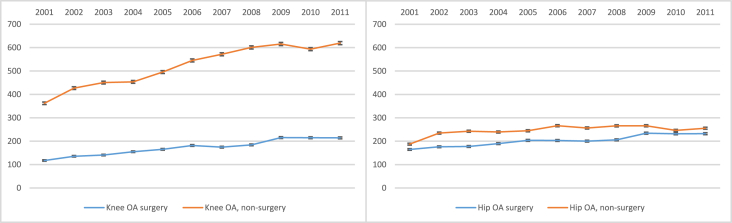
Prevalence per 100,000 people of knee and hip OA surgeries and non-surgery specialist care visits, with 95% confidence intervals, for each year from 2001 to 2011. The numbers reflect the number of people having at least one of the studied outcomes per year.

The prevalence of all outcomes was higher in 2011 in comparison to 2001 (Fig. 1). In 2001 the prevalence (per 100,000 people) of knee OA surgery and non-surgery specialist care visits were 117 (95% CI:114, 120) and 362 (95% CI:357, 367), respectively, while the numbers were 214 (95% CI: 210, 218) and 619 (95% CI: 613, 626) in 2011. The corresponding numbers for hip OA were 165 (95% CI:161, 168) and 187 (95% CI: 184, 191) in 2001, and 232 (95% CI:228, 236) and 255 (95% CI:251, 260) in 2011.

#### 3.1.1 Decomposition of secular trends in prevalence

For knee OA surgery, there were about 97 (96.8, 95% CI: 91.8, 101.8) more surgeries per 100,000 people in 2011 compared with 2001 (Table 2). About 43.6% of this rise in prevalence was attributed to changes in the population characteristics, i.e. explained part of the decomposition, while about 56.4% of the difference was attributed to differences in the coefficients of the sociodemographic characteristics and unmeasured factors, i.e. unexplained part of the decomposition. The explained part was largely made up of differences in age, parents' country of birth, individual country of birth, parents' educational attainment, civil status and individual educational attainment. Differences in the three latter variables, i.e. higher proportion educated parents/less missing information about parents' education, higher proportion of single people and people with higher education, contributed to decline in prevalence, while the difference in the three former, i.e. older age, higher proportion of Sweden-born parents/less missing information about parents' country of birth and higher proportion of foreign-born people, contributed to increase in prevalence. The other variables' contribution was inconclusive and/or negligible. What concerns the coefficient effect, age and educational attainment contributed to increase in prevalence, while country of birth contributed to decline in prevalence. As an example, the results in Table 2 shows that there would have been around 95 more (95.1, 95% CI: 74.4, 115.8) knee OA surgeries per 100,000 people in 2011 if the age coefficient was the same in 2011 as in 2001. Unmeasured factors also accounted for a substantial portion of unexplained part. For knee OA non-surgery visits, there were about 257 (257.1, 95% CI: 248.6, 265.7) more visits per 100,000 people in 2011 compared with 2001. Of this difference, approximately 27.1% could be explained by the population characteristics with age, parents' country of birth, and father's educational attainment having the greatest contributions (Table 2). In general, the patterns of coefficient effects for non-surgery knee OA visits were similar to ones for knee OA surgery.

**Table 2:** Blinder–Oaxaca decomposition results of changes in the prevalence per 100,000 people of knee and hip OA surgeries and non-surgery specialist care visits between 2001 (reference) and 2011. 95% Confidence intervals are shown in parentheses. The number of observations were 10,153,833 and the results are obtained from the same model on the same subjects.

	Knee OA surgery aΔ ​= ​96.8 (91.8, 101.8)		Knee OA, non-surgery aΔ ​= ​257.1 (248.6, 265.7)		Hip OA surgery aΔ ​= ​67.5 (62.0, 73.0)		Hip OA, non-surgery aΔ ​= ​68.0 (62.2, 73.7)	
	Explained b43.6%	Unexplained b56.4%	Explained b27.1%	Unexplained b72.9%	Explained b70.7%	Unexplained b29.3%	Explained b62.9%	Unexplained b37.1%
Total	42.2 (38.9, 45.4)	54.6 (49.2, 60.5)	69.7 (64.4, 75.0)	187.5 (177.9, 197.0)	47.7 (44.2, 51.2)	19.8 (13.9, 25.8)	42.8 (39.2, 46.4)	25.2 (18.9, 31.5)
Constant		−29.0 (−53.0, −4.9)		2.8 (−62.2, 67.8)		−21.3 (−35.7, −6.9)		−18.9 (−43.8, 60.2)
Education (0 ​= ​7 ​yrs)	−7.1 (−9.8, −4.4)	17.6 (7.2, 28.5)	−8.1 (−10.9, −5.3)	50.0 (19.0, 80.9)	4.8 (2.8, 6.8)	7.9 (1.5, 14.2)	3.8 (2.1, 5.4)	11.8 (0.7, 22.9)
Education of mother (0 ​= ​≥10 years of education)	−10.3 (−15.7, −4.9)	−6.2 (−8.6, −3.9)	−1.3 (−6.1, 3.5)	−12.5 (−17.7, −7.4)	−7.5 (−11.8, −3.1)	−4.9 (−6.5, −3.4)	−1.5 (−4.8, 1.7)	−5.4 (−7.5, −3,3)
Education of father (0 ​= ​≥10 years of education)	−18.6 (−24.6, −12.6)	−6.9 (−10.2, −3.7)	−21.2 (−26.2, −16.1)	−8.2 (−15.5, −0.9)	−18.4 (−23.3, −13.5)	−3.4 (−5.3, −1.5)	−8.6 (−11.9, −5.2)	−3.8 (−6.7, −0.9)
Income (0 ​= ​the first quintile group)	−0.0 (−0.1, 0.1)	−0.0 (−0.0, 0.0)	−0.0 (−0.1, 0.1)	−0.0 (−0.0, 0.0)	−0.0 (−0.0, 0.0)	0.0 (−0.0, 0.0)	−0.0 (−0.1, 0.1)	0.0 (−0.0, 0.0)
Employment (0 ​= ​unemployed)	0.1 (−0.0, 0.1)	4.9 (4.2, 5.6)	0.0 (−0.0, 0.1)	9.6 (7.8, 11.4)	0.1 (−0.0, 0.1)	2.3 (1.7, 2.9)	0.1 (−0.0, 0.2)	2.3 (1.5, 3.0)
Civil status (0 ​= ​never married)	−11.7 (−14.1, −9.3)	−0.7 (−2.4, 1.0)	−12.1 (−13.8, −10.5)	−4.8 (−8.9, −0.7)	−4.4 (−5.7, −3.0)	−1.4 (−2.3, −0.4)	−2.6 (−3.6, −1.6)	−0.4 (−1.9, 1.1)
Country of birth (0 ​= ​Sweden-born)	5.8 (4.8, 6.7)	−18.7 (−22.5, −15.0)	10.9 (9.9, 11.9)	−48.8 (−59.2, −38.4)	−0.1 (−1.2, 0.9)	−9.7 (−12.6, −6.8)	1.7 (0.9, 2.4)	−10.2 (−14.2, −6.3)
Country of birth of parents (0 ​= ​both parents born in Sweden)	43.3 (37.8, 48.7)	−1.6 (−4.3, 1.2)	49.0 (45.4, 52.6)	1.1 (−5.7, 8.01)	35.6 (32.1, 39.1)	1.3 (−0.3, 2.9)	20.9 (18.7, 23.1)	2.2 (−0.5, 4.9)
Sex (0 ​= ​female)	−0.2 (−0.3, −0.1)	−0.1 (−0.2, 0.1)	−0.3 (−0.4, −0.2)	−0.1 (−0.3, 0.1)	−0.2 (−0.2, −0.1)	−0.0 (−0.1, 0.0)	−0.2 (−0.2, −0.1)	−0.0 (−0.1, 0.1)
Age (0 ​= ​≥ 35 ​yrs)	40.9 (36.6, 45.3)	95.1 (74.4, 115.8)	52.7 (50.5, 55.0)	198.4 (142.9, 253.8)	37.8 (35.0, 40.5)	49.0 (34.2, 63.9)	29.2 (27.6, 30.8)	47.6 (25.8, 69.4)

a The difference in educational inequalities (measured by Wagstaff index) between 2001 and 2011.

b The relative contribution.

For hip OA, there were about 68 (67.5, 95% CI: 62.0, 73.0) and 68 (68.0, 95% CI: 62.2, 73.7) more OA surgeries and non-surgery visits per 100,000 people, respectively, in 2011 in comparison to 2001. About 70.7% and 62.9% of the difference in hip OA surgery and non-surgery visits, respectively, could be explained by changes in the population characteristics and remaining were attributed to changes in the associations of the population characteristics and unmeasured factors. Concerning the explained part, age, parents' country of birth and father's educational attainment contributed considerably to both hip OA surgery and non-surgery visits, where the latter variable contributed to a decrease in prevalence. What concerns the unexplained parts for hip OA surgery and non-surgery hip OA visits, they were largely determined by age, educational attainment and country of birth, where the latter contributed to a decrease in prevalence (Table 2). Unmeasured factors also contributed substantially to the changes in these outcomes over time.

### 3.2 Changes in educational inequalities

**Fig. 2:**
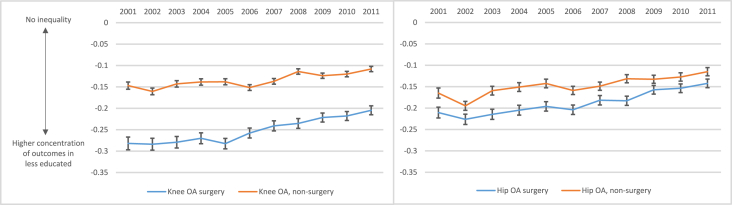
Relative educational inequality, measured by the concentration index (Wagstaff index) with 95% confidence intervals, for each year from 2001 to 2011 for knee OA outcomes (left) and hip OA outcomes (right).

In all study years, the relative concentration indices were negative (values ranged from −0.28 in year 2001 to −0.11 in year 2011) for both knee and hip OA surgery and non-surgery visits indicating that these outcomes were disproportionately concentrated among people with lower education (Fig. 2). The absolute magnitude of the concentration indices for all outcomes were smaller in 2011 compared with 2001 suggesting a decline in educational inequalities in the four OA outcomes. For both knee and hip OA, greater educational inequality was seen for OA surgery than for non-surgery visits with the greater difference for knee than hip OA (Fig. 2).

The contributions of sociodemographic variables to the changes in the relative inequality between 2001 and 2011 are presented in Table 3. The contributions of changes in the population characteristics (explained part) ranged from 15% (knee OA surgery) to 42% (hip OA non-surgery visits). For all outcomes, changes in the population age, civil status, and proportion of people with a foreign background contributed to decrease in relative inequalities, while change in father's educational attainment contributed to increase in relative inequalities (Table 3). Changes in the coefficients of the population characteristics (unexplained part) mostly contributed to increase in relative inequalities, while unmeasured factors contributed substantially to decrease in relative inequalities. Among population characteristics, the changes in the strengths of the associations of age and mother's educational attainment with relative concentration indices had the greatest contributions to changes in relative inequalities over time.

**Table 3:** Blinder–Oaxaca decomposition results of changes in relative educational inequalities (measured by Wagstaff index) in knee and hip OA surgeries and non-surgery specialist care visits between 2001 (reference) and 2011. 95% Confidence intervals are shown in parentheses. The number of observations were 10,153,833 and the results are obtained from the same model on the same subjects.

	Knee OA surgery aΔ ​= ​0.080 (0.062, 0.098)		Knee OA, non-surgery aΔ ​= ​0.038 (0.028, 0.049)		Hip OA surgery aΔ ​= ​0.070 (0.053, 0.086)		Hip OA, non-surgery aΔ ​= ​0.052 (0.036, 0.067)	
	Explained b15%	Unexplained b85%	Explained b34.2%	Unexplained b65.8%	Explained b35.7%	Unexplained b64.3%	Explained b42.3%	Unexplained b57.7%
Total	0.012 (0.004, 0.020)	0.068 (0.049, 0.087)	0.013 (0.008, 0.018)	0.025 (0.014, 0.036)	0.025 (0.017, 0.033)	0.045 (0.027, 0.063)	0.022 (0.014, 0.030)	0.030 (0.012, 0.047)
Constant		0.214 (0.123, 0.304)		0.067 (0.009, 0.124)		0.225 (0.140, 0.310)		0.167 (0.080, 0.252)
Education of mother (0 ​= ​≥10 years of education)	−0.002 (−0.007, 0.004)	−0.013 (−0.024, −0.003)	−0.001 (−0.003, 0.002)	−0.015 (−0.022, −0.009)	0.001 (−0.004, 0.007)	−0.016 (−0.026, −0.006)	0.003 (−0.001, 0.008)	−0.021 (−0.030, −0.011)
Education of father (0 ​= ​≥10 years of education)	−0.010 (−0.015, −0.006)	−0.007 (−0.020, 0.007)	−0.006 (−0.009, −0.003)	−0.007 (−0.016, 0.002)	−0.006 (−0.011, −0.002)	−0.023 (−0.036, −0.011)	−0.007 (−0.012, −0.003)	−0.010 (−0.022, 0.003)
Income (0 ​= ​the first quintile group)	0.000 (−0.000, 0.000)	0.000 (−0.000, 0.000)	0.000 (−0.000, 0.000)	−0.000 (−0.000, 0.000)	0.000 (−0.000, 0.000)	−0.000 (−0.000, 0.000)	0.000 (−0.000, 0.000)	−0.000 (−0.000, 0.000)
Employment (0 ​= ​unemployed)	0.000 (−0.000, 0.000)	−0.000 (−0.005, 0.004)	0.000 (−0.000, 0.000)	−0.002 (−0.005, 0.001)	0.000 (−0.000, 0.000)	−0.000 (−0.005, 0.004)	0.000 (−0.000, 0.000)	−0.002 (−0.006, 0.002)
Civil status (0 ​= ​never married)	0.001 (−0.000, 0.002)	−0.000 (−0.008, 0.007)	0.001 (0.001, 0.002)	−0.002 (−0.006, 0.003)	0.002 (0.001, 0.003)	−0.002 (−0.009, 0.006)	0.001 (0.000, 0.002)	−0.008 (−0.015, −0.001)
Country of birth (0 ​= ​Sweden-born)	0.003 (0.001, 0.004)	0.001 (−0.029, 0.031)	0.002 (0.001, 0.003)	0.014 (−0.003, 0.032)	0.005 (0.004, 0.007)	−0.008 (−0.035, 0.020)	0.005 (0.003, 0.006)	0.009 (−0.018, 0.035)
Country of birth of parents (0 ​= ​both parents born in Sweden)	0.008 (0.003, 0.013)	−0.016 (−0.028, −0.003)	0.005 (0.002, 0.007)	−0.006 (−0.014, 0.003)	0.010 (0.005, 0.015)	0.001 (−0.011, 0.014)	0.010 (0.006, 0.015)	−0.005 (−0.017, 0.007)
Sex (0 ​= ​female)	0.000 (0.000, 0.000)	−0.000 (−0.001, 0.000)	0.000 (0.000, 0.000)	0.000 (0.000, 0.001)	0.000 (−0.000, 0.000)	−0.000 (−0.001, 0.000)	0.000 (−0.000, 0.000)	0.000 (−0.000, 0.001)
Age (0 ​= ​≥ 35 ​yrs)	0.012 (0.010, 0.013)	−0.110 (−0.224, 0.003)	0.012 (0.011, 0.013)	−0.024 (−0.094, 0.046)	0.012 (0.011, 0.014)	−0.132 (−0.236, −0.027)	0.010 (0.008, 0.011)	−0.100 (−0.206, 0.005)

a The difference in educational inequalities (measured by Wagstaff index) between 2001 and 2011.

b The relative contribution.

**Fig. 3:**
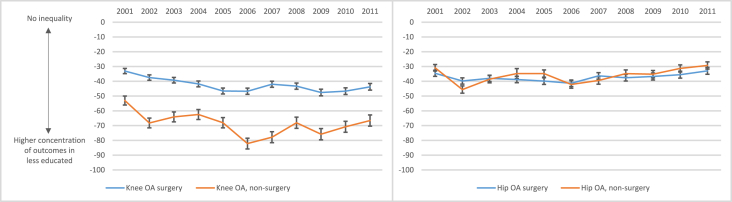
Absolute educational inequality, measured by the Absolute Concentration Index with 95% confidence interval, per 100,000 people for each year from 2001 to 2011 for knee OA outcomes (left) and hip OA outcomes (right).

The absolute educational inequalities in knee OA surgery and non-surgery visits increased from −33 (95% CI: 35, −31) and −53 (95% CI: 56, −50) per 100,000 people in 2001, respectively, to −44 (95% CI: 42, −46) and −67 (95% CI: 70, −63) in 2011 (Fig. 3).

## 4 Discussion

In this nation-wide study, we have examined the changes in prevalence as well as relative and absolute educational inequalities in knee and hip OA surgery and non-surgery specialist care visits in Sweden from 2001 to 2011. Furthermore, we have examined to what extent certain sociodemographic factors can explain these changes. The prevalence of all outcomes rose over time. For knee OA outcomes, this increase was largely attributed to the changes in the strengths of the associations between sociodemographic factors and the outcomes, while for hip OA outcomes changes in the population characteristics had greater contributions to the change in prevalence. We also found that educational inequalities were in favour of more educated people, even though the magnitude of relative inequalities were declining over study period. Conversely, our results showed that the absolute educational inequalities for knee OA outcomes were increasing over time, while these were stable for the hip OA outcomes.

The observed increased prevalence of OA outcomes in the current study is in line with previous research that have predicted an increasing burden of OA [2,30,31]. The unexplained part of the change in prevalence for all outcomes was in large attributed to age and our results suggest a weaker positive association between age and OA outcomes in 2011 than in 2001. The latter finding, that age had a smaller impact on OA outcomes in 2011, is consistent with research that has shown that the OA surgery is not limited to the older population [32]. Previous research has reported that there was an increase in incidence of knee OA surgery in younger patients (<55 years) in Sweden between the late 90s to the time near 2010 [32]. The reason to the increase in younger patients has been suggested to be due to a possible more severe OA in younger population, or a broadening of indication for surgery [32]. Ageing population and a higher proportion of Sweden-born parents between 2001 and 2011 contributed considerably to the increase in prevalence of the hip OA outcomes. Considering age is an important risk factor for OA [1], the finding is not surprising. What concerns the increase in prevalence related to a higher proportion of Sweden-born parents, one may speculate that the finding reflects ethnical differences in hip OA outcomes. For instance, previous research has shown that there exists ethnic differences in the utilization rate of total hip replacement surgery to the extent that foreign patients appear to be less likely to undergo surgery [33]. However, the referred research was conducted in North America and it is questionable whether the results are generalizable to Swedish conditions. In addition, still it is unclear why the variable describing individual country of birth did not contribute considerably. This itself indicates that the variable concerning parents' country of birth represents something more than just ethnicity.

We found a higher concentration of all outcomes in people with lower education in comparison with higher education. This implies that the higher burden of non-surgery specialist care visits in people with lower education was met with higher prevalence of OA surgery in the same group. There was a declining trend in the relative educational inequalities for all outcomes over the study period. At the same time, a trend of increasing absolute educational inequalities for knee OA outcomes were seen from 2001 to 2011, while the absolute inequalities in hip OA outcomes remained unchanged. This finding might reflect a smaller proportional increase in prevalence of the outcomes in low educated compared to high educated. This result may imply a decrease in access to specialist care among people with lower education compared to people with higher education. Whether this possible decrease in access to specialist care is medically motivated due to, for instance, better management of OA at primary care or due to a true lower prevalence of OA in this group is unknown.

Our findings regarding higher concentration of all outcomes in people with low education are in line with previous studies that have reported an inverse association between educational attainment and rate of knee arthroplasty due to OA [17] and prevalence/symptoms of knee [[9], [10], [11], [12], [13], [14]] and hip OA [[14], [15], [16]]. The higher concentration of the outcomes among people with low educational attainment is probably a result of many factors combined. In comparison to high educated people, people with low level of education are more likely to have a physically demanding occupation, which has been suggested as a risk factor for knee [34,35] and hip OA [36]. There might also be educational differences in severity of OA and thus also differences in health care seeking as a result. For instance, several studies have found a higher pain prevalence and risk of chronic pain as well as lower health-related quality of life for various medical conditions including knee OA, in people with lower socioeconomic status in general [11,37,38] and lower educational attainment in specific [11,39,40]. It is also possible that the treatment that the patient is given and/or is willing to accept differs between educational groups. A study conducted in Sweden reported that individuals participating in the OA self-management program *Better Management of Patients with OsteoArthritis* were generally more educated compared to the general population, indicating that the program might reach people with low level of education to a lesser extent [41]. It should also be noted that the education-health associations might be due to selection, i.e. people with better health at early life tend to attain higher education and have better health (e.g. lower risk of OA) later in life. For instance, it has been suggested that low birth weight and prematurity is associated with lower educational attainment [42] as well as hip, but not knee, arthroplasty due to OA [43].

The results from the decomposition analyses regarding educational inequalities suggest that a considerable part of the observed decrease in relative educational inequalities between 2001 and 2011 is due to unmeasured factors suggesting that there exist important factors that we have not accounted for. In general, the changes in the population characteristics in terms of age, proportion of individuals with foreign background (higher proportion of foreign-born), and father's educational attainment (higher proportion of low education) between 2001 and 2011 seem to be important in explaining the change in educational inequalities in knee and hip OA outcomes. For all outcomes, the coefficient of parents' educational attainment predicted increases in relative inequality. That the parents educational attainment is of importance when studying educational inequalities is not surprising since it is well-known that parents' educational attainment is associated with their child's educational attainment [44].

Better management of patients with OsteoArthritis was initiated in 2008 and since 2010 it has been a National Quality Register evaluating a standardized OA management program (https://boa.registercentrum.se). When examining the graphs concerning absolute and educational inequalities for the outcomes we could not notice any break in the trend. However, the program was initiated in the later part of our study period and thus not fully implemented during the studied time. Thus, it would be interesting to study prevalence and educational inequalities of OA outcomes during the time period after the program was more widely implemented in Sweden.

The major strength of our study lies in the large, nation-wide study sample and use of individual-level data. The decomposition analysis offers a novel insight into OA research, since decomposition analysis has not, as far as we know, been employed in the OA research field. However, several limitations of the current study need to be considered. First, due to the lack of national primary care data, our estimations are based on information from the specialist care and it is unknown what the true prevalence (OA in primary care included) of diagnosed OA by different educational groups were during the time period. Hence, our results might not be generalizable to the overall prevalence of OA in the general population. Secondly, for knee OA only a relatively small part of the change in prevalence and educational inequalities of the outcomes could be attributed to the ‘explained’ component, i.e. sociodemographic determinants measured and included in our models Unmeasured factors contributed substantially to the “unexplained” component. This finding suggests a need for collection or linkage of data on other potentially important determinants in future studies. These may include lifestyle or behavioural factors or measures of disease severity and health status. However, the standardised collection and recording of these time-varying factors within routine datasets is typically challenging. Thirdly, even though the reliability of the National Patient Register is considered to be good and reflect the data available in the regional patient administrative systems [45], problems with data reporting, mainly by private healthcare providers, have been reported [45]. Fourthly, the variables representing the parents' education and country of birth had substantial missing data, which will weaken the generalizability of the results. In addition, it is possible that the observed increases in the OA outcomes are partly attributed improved diagnostic coding of OA. This since it has been noted that the coverage of the patient register has improved over time [45].

In conclusion, the prevalence of knee and hip OA surgery and non-surgery specialist care visits increased from 2001 to 2011 in Sweden. For knee OA outcomes, this increase was mainly due to changes in the strength of the associations between sociodemographic factors and OA outcomes. With respect to hip OA outcomes, the increase was mainly due to changes in age and parents’ country of birth over time. There was a decreasing trend in relative educational inequalities for all outcomes over time while the absolute inequalities were decreasing or stable. This may imply a decrease in access to specialist care among people with lower education compared to people with higher education. Future research should collect data on additional variables, since only a small portion of the change in prevalence of knee OA outcomes and educational inequalities of the knee and hip OA outcomes could be explained by the characteristics included in the study. Our study provides novel insight in the study of trends in prevalence and educational inequality in knee and hip OA, which in the long term is important in order to be able to implement preventative measures against the disease and to reduce inequalities.

## Author contributions

ML and AK contributed to the design of the study and interpretation of data. ML contributed to analysis of the data and wrote the first draft of the manuscript. GP and ME contributed to interpretation of data. All authors revised the manuscript for important intellectual content and approved of the final version to be submitted. ML (maria.lindeus@med.lu.se) takes responsibility for the integrity of the work as a whole.

## Role of funding source

This study was supported by grants from *The Swedish Research Council*, *The Swedish Rheumatism Association*, *Governmental Funding of Clinical Research within National Health Service (ALF)*, *Region Skåne, Lund University, Greta and Kocks Foundation*, *Österlund Foundation, King Gustaf V's 80*th *Birthday Foundation*, *Foundation for Persons with Movement Disability in Skåne* and *Crafoord Foundation*. The funder had no role in study design, data collection and analysis, or preparation of the manuscript.

## Conflict of interest

The authors declare no conflict of interest.
