# Supplementary material for: Changes in educational inequalities in knee and hip osteoarthritis surgery and non-surgery specialist care visits over time in Sweden
Source: Osteoarthr Cartil Open. 2024 Apr 11;6(2):100470. doi: 10.1016/j.ocarto.2024.100470 (PMC11053214; doi:10.1016/j.ocarto.2024.100470)
Supplement: Multimedia component 1 [file mmc1.docx]

**Supplementary material**

Table A1, Definition of years of education

| Statistics Sweden’s  SUN 2000 | Statistics Sweden’s description of the code | Years of education in the study |
| --- | --- | --- |
| <200 | Pre-secondary education shorter than 9 years | 7 |
| ≥200 and <300 | Pre-secondary education of at least 9 years | 9 |
| >300 and <320 | Secondary education of at least one semester (but not 2 years) | 10 |
| ≥320 and <330 | Secondary education at least 2 years (but not 3 years) | 11 |
| ≥330 and <400 | Secondary education of 3 years | 12 |
| ≥410 and <500 | Postsecondary education of at least one semester (but not 2 years) | 13 |
| ≥520 and <530 | Postsecondary education of at least 2 years (but not 3 years) | 14 |
| ≥530 and <540 | Postsecondary education of at least 3 years (but not 4 years) | 15 |
| ≥540 and <550 | Postsecondary education of at least 4 years (but not 5 years) | 16 |
| ≥550 and <600 | Postsecondary education of 5 years or longer | 17 |
| ≥600 and <640 | Other / unspecified research education and Licentiate degree | 18 |
| 640 | PhD education | 20 |

Information about Statistics Sweden´s SUN 2000 variable: <https://www.scb.se/contentassets/aeeedec0e28c465aa524429407dcd5ba/mis-sun-2000.pdf>

Table A2. Surgery codes used for knee and hip OA surgery

| Knee OA surgery | |
| --- | --- |
| NGB49 | Primary total prosthetic replacement of knee joint using cement |
| NGB19 | Primary partial prosthetic replacement of knee joint using cement |
| NGK59 | Angulation, rotation or displacement osteotomy of knee or lower leg |
| NGB29 | Primary total prosthetic replacement of knee joint not using cement |
| NGB59 | Primary prosthetic interposition arthroplasty of knee joint |
| NGB53 | Primary patellofemoral prosthesis |
| NGB09 | Primary partial prosthetic replacement of knee joint not using cement |
| NGB39 | Primary total prosthetic replacement of knee joint using hybrid technique |
|  |  |
| Hip OA surgery | |
| NFB49 | Primary total prosthetic replacement of hip joint using cement |
| NFB29 | Primary total prosthetic replacement of hip joint not using cement |
| NFB39 | Primary total prosthetic replacement of hip joint using hybrid technique |
| NFB99 | Other primary prosthetic replacement of hip joint |
| NFK59 | Angulation, rotation or displacement osteotomy of femur |
| NFB62 | Primary prosthetic replacement of joint surface of femoral head |

|  | 2001 | 2002 | 2003 | 2004 | 2005 | 2006 | 2007 | 2008 | 2009 | 2010 | 2011 |
| --- | --- | --- | --- | --- | --- | --- | --- | --- | --- | --- | --- |
| Number of people (n) | 4,794,693 | 4,864,808 | 4,925,960 | 4,979,842 | 5,031,336 | 5,091,331 | 5,150,333 | 5,205,573 | 5,265,630 | 5,314,087 | 5,359,186 |
| Number of knee OA surgeries (n) | 5,628 | 6,433 | 6,935 | 7,716 | 8,308 | 9,248 | 8,989 | 9,588 | 11,339 | 11,408 | 11,477 |
| Number of hip OA surgeries (n) | 7,895 | 8,568 | 8,743 | 9,442 | 10,248 | 10,342 | 10,322 | 10,717 | 12,332 | 12,317 | 12,442 |
| Number of non-surgery visits in specialist care with knee OA as main diagnosis (n) | 17,360 | 20,758 | 22,195 | 22,583 | 24,929 | 27,757 | 29,432 | 31,274 | 32,408 | 31,535 | 33,184 |
| Number of non-surgery visits in specialist care with hip OA as main diagnosis (n) | 8,988 | 11,419 | 11,953 | 11,922 | 12,308 | 13,554 | 13,682 | 13,829 | 14,012 | 13,090 | 13,688 |
| Mean age ± SD | 55.8 ± 13.9 | 55.9 ±  14.0 | 56.1 ±  14.1 | 56.3 ±  14.1 | 56.4 ±  14.2 | 56.6 ±  14.2 | 56.7 ±  14.3 | 56.8 ±  14.3 | 56.9 ±  14.3 | 57.1 ±  14.4 | 57.3 ± 14.4 |
| Females (%) | 48.6 | 48.7 | 48.7 | 48.7 | 48.7 | 48.7 | 48.8 | 48.8 | 48.8 | 48.8 | 48.9 |
| Foreign-born (%) | 12.8 | 13.0 | 13.2 | 13.4 | 13.6 | 13.9 | 14.2 | 14.5 | 14.9 | 15.2 | 15.6 |
| Missing foreign-born (%) | 0 | 0 | 0 | 0 | 0 | 0 | 0 | 0 | 0 | 0 | 0 |
| Sweden-born parents (%) | 57.0 | 58.0 | 58.9 | 59.7 | 60.5 | 61.3 | 62.0 | 62.7 | 63.3 | 63.9 | 64.4 |
| Missing (%) | 34.1 | 33.0 | 31.9 | 31.2 | 29.9 | 28.9 | 28.1 | 27.2 | 26.4 | 25.7 | 25.0 |
| Educational attainment |  |  |  |  |  |  |  |  |  |  |  |
| > 12 years of education (%) | 25.6 | 26.3 | 26.9 | 27.5 | 28.1 | 28.7 | 29.4 | 30.1 | 30.8 | 31.5 | 32.2 |
| Missing (%) | 0 | 0 | 0 | 0 | 0 | 0 | 0 | 0 | 0 | 0 | 0 |
| Educational attainment of mother |  |  |  |  |  |  |  |  |  |  |  |
| <10 years of education (%) | 30.6 | 30.9 | 31.2 | 31.5 | 31.7 | 31.8 | 31.9 | 31.9 | 31.9 | 31.9 | 31.9 |
| Missing (%) | 51.7 | 50.1 | 48.7 | 47.4 | 46.1 | 44.8 | 43.6 | 42.4 | 41.2 | 40.1 | 39.1 |
| Educational attainment of father |  |  |  |  |  |  |  |  |  |  |  |
| <10 years of education (%) | 20.2 | 20.8 | 21.4 | 21.8 | 22.3 | 22.7 | 23.0 | 23.4 | 23.7 | 24.0 | 24.2 |
| Missing (%) | 64.3 | 62.7 | 61.3 | 59.8 | 58.4 | 57.0 | 55.7 | 54.3 | 53.0 | 51.8 | 50.7 |
| Income |  |  |  |  |  |  |  |  |  |  |  |
| Mean disposable income (SEK) | 163,276 | 170,253 | 173,573 | 178,599 | 188,320 | 172,556 | 219,520 | 223,671 | 227,283 | 234,738 | 244,115 |
| Missing (n) | 45 | 47 | 51 | 63 | 66 | 83 | 92 | 104 | 115 | 64 | 1 |
| Civil status |  |  |  |  |  |  |  |  |  |  |  |
| Married (%) | 56.7 | 55.9 | 55.2 | 54.7 | 54.3 | 53.9 | 53.7 | 53.5 | 53.2 | 53.1 | 52.9 |
| Previously married (%) | 24.6 | 24.6 | 24.7 | 24.7 | 24.7 | 24.6 | 24.5 | 24.3 | 24.2 | 24.1 | 24.1 |
| Never married (%) | 18.8 | 19.5 | 20.1 | 20.6 | 21.0 | 21.5 | 21.9 | 22.2 | 22.6 | 22.8 | 23.1 |
| Missing (%) | 0 | 0 | 0 | 0 | 0 | 0 | 0 | 0 | 0 | 0 | 0 |
| Employment |  |  |  |  |  |  |  |  |  |  |  |
| Employed (%) | 58.0 | 58.5 | 57.6 | 58.5 | 58.3 | 58.7 | 59.4 | 59.1 | 57.8 | 58.0 | 58.0 |
| Missing (%) | 0 | 0 | 0 | 0 | 0 | 0 | 0 | 0 | 0 | 0 | 0 |

Table A3. Descriptive statistics of the study populations of the years 2001 to 2011
